# Supplementary figures and images for: An ACE2 decoy can be administered by inhalation and potently targets omicron variants of SARS‐CoV‐2
Source: EMBO Mol Med. 2022 Sep 23;14(11):e16109. doi: 10.15252/emmm.202216109 (PMC9539395; doi:10.15252/emmm.202216109)

Expanded View Figures

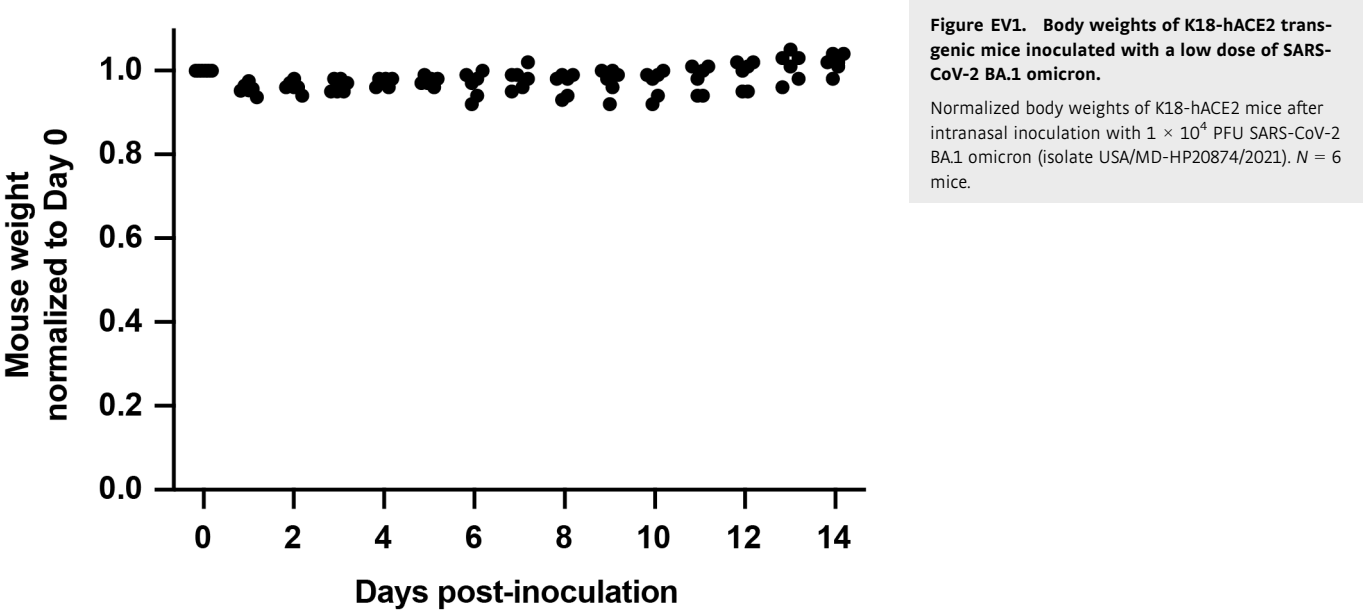

Supplement: Supplementary file 1 — Expanded View Figures PDF [file EMMM-14-e16109-s004.pdf]
